# What Is New about the Exposome? Exploring Scientific Change in Contemporary Epidemiology

**DOI:** 10.3390/ijerph17082879

**Published:** 2020-04-22

**Authors:** Stefano Canali

**Affiliations:** Institute of Philosophy, Leibniz University Hannover, Im Moore 21, 30167 Hannover, Germany; stefano.canali@philos.uni-hannover.de

**Keywords:** exposome, scientific change, postgenomics, scientific epistemology

## Abstract

In this commentary, I discuss the scientific changes brought by the exposome, asking what is new about this approach and line of research. I place the exposome in a historical perspective, by analyzing the conditions under which the exposome has been conceived, developed and established in the context of contemporary epidemiological research. I argue that the exposome has been developed by transferring approaches, methods and conceptualizations from other lines of research in the life and health sciences. I thus discuss the conceptual and methodological innovations of the exposome as a result of the merging and adaptation of these elements for new uses and purposes. On this basis, I argue that the novelty of the exposome should be seen in incremental rather than revolutionary terms and, in this sense, the exposome shares significant elements with other projects and repertoires in postgenomics. I conclude by discussing the consequences of this analysis for the potential limitations and future development of exposome research.

## 1. Introduction

In the last decade, the exposome has emerged as a new concept and approach in epidemiological and biomedical research more generally and particularly to characterize the totality of environmental exposures, horizontally and vertically. As such, in epidemiology the exposome approach is considered and presented as highly innovative [1]. First, while traditionally epidemiologists have focused on the external level of exposure, the exposome aims at expanding this approach by distinguishing between different levels of exposure and focusing on the internal component of exposure, which is considered a type of exposure as important as the external ones. Second, the simultaneous study of different levels of exposure and chemicals is considered particularly innovative too, especially in comparison to the traditional focus on single chemicals or single types of exposure. Third, the all-encompassing approach of the exposome is also a way of implementing a life course and dynamic approach, thus moving away from measurements at single points and towards an understanding of issues of exposure and disease as developing dynamically throughout a lifetime. 

In the scientific literature, the term “paradigm” is often used to discuss the strongly innovative character of the exposome. For instance, the exposome has been presented as “a new and exciting paradigm for the improvement and integration of currently scattered and uncertain data on the environmental component in disease etiology” [2]; an “operational paradigm” for exposure science [3]; a “paradigm shift” for public health [4]; a new “research paradigm” for environmental epidemiology [5], planetary health, biomonitoring and next-generation exposure assessment [6,7,8]. Paradigm, as a term connected to discussions and analyses of scientific change and innovation, was popularized in the history, philosophy and sociology of science by Thomas Kuhn [9]. Kuhnian paradigms are the “shared commitments of a scientific group” [10], i.e., the concepts, theories, assumptions, generalizations, values, exemplary problems and solutions, which provide a specific identity and constitution to research communities. While the use of this term to characterize the innovation of the exposome with a generic and relatively loose meaning could be justified, in this commentary I will argue that it does not recognize the complex and nuanced type of innovation of the exposome, which is actually in continuity with longstanding and contemporary approaches in biomedical research. This commentary looks back at the recent history of the exposome and proposes a view of the innovation of the exposome that explains its role in contemporary research, whilst acknowledging limitations and possible future developments.

## 2. The Lineage of the Exposome 

An analysis of the development and establishment of the exposome shows its conceptual, methodological and material connections to the wider research context of the life and health sciences and in particular three main approaches and lines of research: the sequencing repertoire, biomarkers research and exposure science. 

The exposome was famously introduced in 2005 by Christopher Paul Wild, who characterized it as a way of describing the totality of environmental exposures that individuals are exposed to in their life course. The exposome “encompasses life-course environmental exposures (including lifestyle factors), from the prenatal period onwards”, as a “highly variable and dynamic entity that evolves throughout the lifetime of the individual” [11]. When Wild introduced the concept, he situated it in the context of several debates in the life and health sciences. In particular, he aimed to shift attention to the “need for methodologic developments in exposure assessment” and for “methods with the same precision for an individual’s environmental exposure as we have for the individual’s genome” [11]. Namely, Wild praised the development made in genomics but also noted how the low penetrance of genetic variants—as opposed to their high prevalence—implies that their contribution to disease burden is crucially linked to the presence of some environmental exposure and therefore argued for a broader consideration of the environmental side. In Wild’s intentions, the exposome was to bring propositions together for epidemiology in a similar way to what the genome had done for genetic research. The year 2005 was just a few years after the end of the Human Genome Project, which officially ran between 1990 and 2003. The project was a major breakthrough for the life sciences, with significant impact on funding, the conceptualization of genes and genetics, science management, technology, data infrastructures, etc. [12]. The exposome has drawn significantly on the repertoire that emerged in the context of genomic projects and has since then increasingly spread in the life and health sciences. 

Conceptually, the exposome lies in a two-fold relation with the genome. On the one hand, the various epistemic breakthroughs of genome-sequencing are constantly mentioned in discussions on the exposome and the wording itself indicates a close relation to the genome. On the other hand, the idea of the exposome as the necessary complement of the genome puts it into a critical position to the genome and points to the need for new and different approaches, beyond genomics. This is evident in many considerations about the low penetrance of genetic variants and their contribution to disease risks and the need for more emphasis on the role of environmental exposure, in contrast to gene-centrism [1]. Whilst retaining a critical perspective on genomic approaches, the hope is that the exposome can do for environmental epidemiology what the genome did for genomics, i.e., to collect and organize under an umbrella concept various ideas and approaches to the study of the relation between environmental exposure and disease. This close relation between the exposome and the sequencing repertoire is also shown by the crucial role of material and institutional elements transferred from the sequencing repertoire. Moreover, from a technical point of view, the exposome heavily relies on omics techniques, whose development can be traced back to sequencing and mapping technologies. To a large extent, the exposome is about bringing the developments in omics techniques from genomics to epidemiological research, as a way of trying to get a similar level of precision in measuring the presence, variation and impact of exposures [3]. 

The sequencing repertoire has been crucial in the development of the exposome from a material and methodological perspective, leading to the conceptualization of an environmental equivalent to the genome and the broadening of the concept of exposure to make use of omics data. Yet the broadening of exposure conceptualized by the exposome was also made possible by the interactions with a second area of research: exposure science. While its roots are connected to early work in industrial and occupational hygiene, exposure science can be considered a relatively new field of research, whose definition and specification took place in the US context around the time of Wild’s initial proposal of the exposome [13,14]. Until the 1920s exposure scientists and environmental epidemiologists collaborated on the study of workplace exposures in the context of occupational disease. This changed with the establishment of two different agencies in the US in the 1970s, which created two distinct paths of research in the field [15]. At the same time, the focus shifted from human health towards risk assessment and compliance with standards, from measurement of personal and individual exposure to predictions of exposure levels and presence of chemicals and toxicants from deterministic models [16]. Still, exposure science has been very important for the exposome, especially from a conceptual point of view, as it has provided the conceptual and methodological tools to look at internal exposure as a type of exposure, which is to be considered one of the components of the exposome and investigated as a type of environment. 

At a more concrete level, exposome research is often carried out by searching for biomarkers of exposure and biomarkers of disease and, thus, studying and analyzing their associations. Biomarkers science is another line of research from which the exposome has transferred significant components, especially at a methodological level. In a broad sense, any medical sign is a biomarker, including measurements such as pulse, blood pressure and more or less sophisticated blood tests; thus, biomarkers can be any indication of any medical state. The use of biomarkers has had a steady increase since the 1980s, especially in the context of clinical trials, as surrogate outcomes of diseases including cancer and heart disease, in basic and clinical drug research [17]. In this sense, the development of molecular techniques and their application and use in the context of biomedical research has played a crucial role, specifying the notion of biomarkers in a research context and pushing the level of abstraction to the molecular level [18]. The connection between biomarkers and the exposome is related to a wider movement in epidemiology, referred to as “molecular epidemiology”, i.e., the drive towards improved quantification of exposure through molecular techniques and measurements [19]. In the exposome context, molecularization has been interpreted as a push for more accuracy and precision at the external and environmental level too and therefore new sources of environmental data, such as Geographical Information Systems, are used to get to a similar microscopic scale and precision level [20]. In addition, the biomarkers approach is often considered different from sequencing methods such as GWAS, as it should enable more direct analyses of what lies in between associations and shed light on molecular pathways and mechanisms [21]. In this way, the approach extends its influence from methodological to theoretical aspects, as the biomarkers perspective has informed etiological approaches and pushed for molecular conceptualizations of health and disease [22].

## 3. The Innovations of the Exposome as an Emerging Research Repertoire 

The exposome has thus historically emerged as a result of the transfer of conceptual, methodological and technical components from three main areas of research, which span from the spectrum of research in the life and health sciences. The transfer of these components into epidemiology and the development of the exposome counts as scientific innovation in itself, in particular because this transfer has implied a new contextualization, repurposing and significant variation of these components. 

First, the transfer of omics techniques from sequencing repertoires and their use as ways to measure the exposome has led to a different interpretation of the evidential value of omics data. One of the currently active areas of work in the exposome approach concerns the repurposing and advancement of omics techniques as untargeted methods, which are used to collect untargeted omics data for statistical analysis. For example, the untargeted use of omics techniques to quantify molecules at the level of intermediary functioning of the metabolism (metabolomics) and proteins more generally (proteomics) is expected to maximize the number of molecules that can be studied and measured [23]. This is happening at the same time as the advancement towards untargeted methods in areas such as high-resolution mass-spectrometry, which is now used to detect and quantity very large quantities of small molecules in blood samples retrieved from longitudinal studies [24]. This use of omics is related to other methodological applications and conceptual interpretations of the exposome, in particular what is referred to as the top-down approach [3]. More generally, this is a way of repurposing the methodological and material component of sequencing repertoires to the context of epidemiological studies. With a narrative that has been crucial for the exposome since its conception, this use of omics is a way to both change the epidemiological landscape and complement genomics, whilst at the same time extending genomic solutions to different fields. The goal with untargeted methods is indeed to conduct exposome-wide association studies (EWAS) to complement genome-wide association studies (GWAS) [25]. 

This use of omics also influences the employment of biomarkers in exposome research, the second component that I have identified in the previous section. In particular, the transfer of the conceptual and methodological tools of biomarkers from the life sciences implies the use of concepts such as internal exposure. This notion has traditionally been used in biomarker science to discuss measurements of the concentration of external, environmental agents and chemicals, for example in blood. The transfer of the notion in epidemiological research allows to expand the concept of exposure, which is reframed to allow different dimensions: within the exposome, to be exposed is to be exposed at different external and internal levels. This is a difference from traditional epidemiology, where historically the notion of exposure is used to discuss external exposure [2]. At the same time, however, the use of biomarkers for exposome research and the transfer of the concept of internal exposure implies a partial shift in the application of biomarkers methodology. In the context of the exposome, internal exposure refers to concentrations of molecules involved in both intermediate metabolism and endogenous processes (e.g., oxidative stress, inflammation), which can be affected by an exposure to an environmental chemical and thus be correlated to external exposure. This way of employing biomarkers methodology is significant for the conceptualization of the relation between external and internal exposure and their boundaries. Hence, this transfer from biomarkers science implies an expansion of the notion of exposure at both ends of the spectrum: exposure can also be measured and take place at the internal level, which is new from an epidemiological perspective; and internal exposure can also be used to study various processes and molecules, which is new from a biomarkers perspective. These conceptual approaches to exposure are currently interpreted with approaches drawn from biomarkers and molecular epidemiology, such as the meet-in-the-middle approach [22]. According to this approach, the goal of the analysis of associations between biomarkers of exposure and biomarkers of outcomes of interest (e.g., disease) is to identify intermediate biomarkers, which potentially link causally exposure and the development of disease [26,27]. 

The interrelations and connections between elements transferred from elsewhere and their repurposing is also evident for exposure science. In this case, what is particularly significant of recent lines of work that are currently active in exposome research is the study of mixtures of exposures. The role of complex mixtures of the substances, environmental agents and pollutants that individuals are exposed to is considered highly significant for the study of the incidence and development of health and disease in populations. One of the goals of the exposome, as a dynamical and all-encompassing approach, is to target these mixtures, which potentially can be correlated with each other as well as other environmental agents and stressors. In the last few years, various statistical approaches have been conceived to study mixtures and their health effects, more specifically, by developing methods to investigate both the cumulative and joint effects of mixtures and their independent role [28]. In the context of the exposome approach, these tools have been applied as a way of measuring and studying parts of the exposome and moving beyond single-pollutant models, whereby “it is not clear if an observed association reflects the effect of the analyzed pollutant or if it acts as a surrogate for another pollutant possibly originating from the same source” [29]. These methods have been transferred and adapted from both exposure science and the sequencing repertoire and have been repurposed in the exposome context to analyze multipollutant cases and multiple correlated exposures. In this sense, the use of these transferred methods is significant because it is connected with the aforementioned shift towards the blurring of internal and external exposures and environment and the increasing push for integrated approaches. 

Therefore, the transfer of conceptual, methodological and material elements from other areas of research in the life and health sciences in the exposome approach has resulted in the repurposing and variation of these elements. In this sense, these repurposed components constitute the main elements of what can be considered as the “exposome repertoire”, i.e., the set of questions, approaches, tools and assumptions that characterize exposome projects. Discussing the exposome as a repertoire emphasizes the complexity of the exposome as an emerging line of research and research program and community in epidemiology. The exposome includes an assemblage of components at various levels, not just conceptual and methodological levels [30], which are both performed when applying the exposome approach and they affect the ways in which the boundaries of the area are determined and newcomers are trained [31]. On this basis, as I discuss in the next section, the exposome is not simply a theoretically and methodologically cemented notion—a paradigm—and does not involve dramatic or paradigmatic shifts to the ways in which epidemiological research is conducted. 

## 4. Scientific Change in the Postgenomic Era: Innovation and Continuity

The analysis of the lineage of the exposome and various areas and approaches that have led to its development and establishment shows how the material, methodological and conceptual components of the exposome have been transferred, merged and adapted from other areas and traditions in the life and health sciences. It is in this sense that I argue that the exposome has been developed in strong continuity with various, well-established approaches in biomedical research and does not constitute the revolutionary and dramatic change of Kuhnian paradigms. However, to say that the exposome is not a new Kuhnian paradigm does not mean that the exposome is not innovative. As we have seen, the transfer of conceptual, methodological and material components from other lines of research in the life and health sciences has led to the repurposing and alignment of these components for a new context. In turn, this has also influenced the application of the exposome approach and has contributed to the shaping of its conceptual and methodological innovations. The innovations of the exposome are particular evident in what counts as exposure and environment and how they are conceptualized and operationalized. The use of omics, mixtures and untargeted mass-spectrometry have contributed to the shaping of the notion of environmental exposure in the exposome, to include exposure in the internal and external environment, at the individual and population level and at different points throughout the lifetime of an individual. This is a conceptual novelty for epidemiological research and in the exposome is operationalized through the transfer of notions such as internal exposure from biomarkers research and methodological and technological approaches from exposure science and sequencing repertoires. The innovation we are seeing as a result of these processes is difficult to attribute precisely to either the introduction of the exposome or the developments in other areas of research. Similarly, the exposome has brought an innovative understanding of the relations between the external and internal environment and what delimits them. First, the emphasis put by the exposome on a plurality of environments is important for the rhetorical position of the exposome as a critical and complementary notion of the genome. Secondly, in the framework of the exposome, the use of new sources on data on environmental exposure at various levels blurs the limits of different types of environment and presents a pluralistic approach to the environment, which is to be investigated as a whole. This pluralistic approach of the exposome is not a radical innovation but rather a continuation of the various ways in which the environment has been conceptualized in epidemiology. Yet, it is also a way of innovatively giving grounding and specification to discussions on what counts as environment and how to investigate its causal role on health and disease, which are increasingly important in postgenomics. 

The term postgenomic has been used to describe the aftermath of the completion of the Human Genome Project and to describe it as a new, revolutionary era in the life and health sciences. The revolutionary view of postgenomics has been criticized by life and health scientists, as well as sociologists, historians and philosophers of science, who have cautioned against claims depicting postgenomics as a new paradigm and a revolution in the sciences, pointing to the continuity between the genomic and postgenomic era [32]. The exposome shares many features with postgenomic projects, insofar as it is based on genomic and genomic-based technologies but is also increasingly aware of the complexity in interpreting genomics data and has a critical engagement with gene-centric approaches [33]. Critiques of the extensive use of genomics data and gene-centrism to explain human disease are constantly present in introductions and presentations of the exposome: the exposome is often discussed as a “post-genome notion”, in the sense that it pushes for research that should provide solutions that the genome did not deliver; yet, exposome research extensively employs omic techniques, which are based on technological developments of the genomics era. In other words, like other postgenomic programs, the exposome is supposed to go beyond genomics—yet it is relies on genomic-based solutions at a methodological and material level. In this sense, the change and innovation of the exposome is similar to the innovation we are seeing in postgenomic more generally. Innovation lies in the different ways in which elements of other areas of research are readapted and merged for new uses. In addition, many of these components are not conceptual: in the postgenomic era, innovation is often the result of the role of non-conceptual and particularly material, performative and technological components, which are crucial as enablers of scientific change. 

In this sense, the exposome is one of the innovative notions, research programs and repertoires that populate the diverse and multifaceted landscape of contemporary postgenomics. The shared elements between the exposome and other postgenomic repertoires do not only speak for the innovative character of the exposome but also for the potential limitations and future developments of the exposome. Lessons from the recent development and success of other research repertories suggest that, to establish stable innovation and success, the development of social and technological infrastructures and the long-term commitment of funding institutions is crucial [26]. In this sense, the development of exposome repositories and databases and the commitment of crucial funding bodies such as the European Research Council and the European Commission, who established dedicated funding tracks in Horizon 2020, are significant. Yet most of the projects funded through these schemes are short-term and lack the blue-skies funding dedicated to other postgenomic projects such as sequencing repertoires. This has created various issues to exposome projects, which often run out of funding and time without having finished the analysis of all the collected data. In addition, the extensive use of genome- and sequencing-based techniques, omics data and molecular biomarkers runs the risk of reinforcing elements of the reductionist approach of molecular tools, by reducing macroscopic phenomena at the individual level and making it more difficult to investigate phenomena at the social level [34]. Molecular and omics data offers an exciting opportunity for exposome research, but its validity and relevance for epidemiological research is not necessarily intrinsic and should be considered in the context of the goals and values of specific research projects [35].

Therefore, as I have tried to show throughout the article, the exposome is an innovative approach for epidemiological research, whose novelty is to be found in the transfer of conceptual, methodological and material components from other areas of research, their adaptation to a new context and the consequent development of new conceptualizations and operationalizations of key notions such as exposure and environment. This has led me to argue that what is new about the exposome is not a radical departure from other longstanding and well-established approaches in the life and health sciences. In other words, I have argued that the exposome is not a new, Kuhnian paradigm. I agree that significant elements of the current research and debate on the causal impact of the environment on health and disease can be framed as elements of a “crisis” that the exposome could solve [36]. Yet, the new solutions proposed by the exposome are not radically new and, perhaps more importantly, they exist in a plurality of other approaches and concepts used to develop and conduct epidemiological research in postgenomics. Thus, currently the exposome does not seem to be completely substituting these other approaches nor does it seem to be incommensurable with epidemiology or environmental health sciences—traits that are characteristic of the paradigmatic change analyzed by Kuhn [9]. The exposome is one of many approaches used and applied in epidemiology, which is an increasingly pluralistic landscape of research [37]. At the same time, as we have seen, this does not mean that the exposome is not innovative. In recent years, philosophers and sociologists of science have argued for different ways of characterizing change and novelty in scientific research [38]. According to these accounts, innovations as a result of transfer should be considered a form of scientific change in itself [31]. In addition, the ways in which scientific change can manifest itself are not necessarily dramatic or paradigmatic and do not have to lead to the radical substitutions or incommensurability [39]. Rather, scientific change is also a form of pluralization, contributing to the expansion and broadening of the plurality of approaches, tools and methods used to investigate phenomena.

## 5. Conclusions

In this commentary, I have analyzed the innovative character of the exposome by discussing the historical and scientific background of its development and in the context of other approaches and lines of research in contemporary biomedical research. I have argued that the innovation of the exposome is not radical and disruptive but should be seen in significant continuity with other approaches in the field and beyond. In this sense, I have also argued that the exposome is close to other projects and repertoires in current postgenomics, which share with the exposome a similar incremental type of innovation. These considerations suggest possible directions and limitations for the sustenance and future development of exposome research. 
